# Effect of Speech Degradation on Top-Down Repair: Phonemic Restoration with Simulations of Cochlear Implants and Combined Electric–Acoustic Stimulation

**DOI:** 10.1007/s10162-012-0334-3

**Published:** 2012-05-09

**Authors:** Deniz Başkent

**Affiliations:** 1 Department of Otorhinolaryngology/Head and Neck Surgery, University Medical Center Groningen, PO Box 30.001, 9700 RB Groningen, the Netherlands; 2 Graduate School of Medical Sciences, Research School of Behavioural and Cognitive Neurosciences, University of Groningen, Groningen, the Netherlands

**Keywords:** phonemic restoration, speech perception, auditory scene analysis, top-down and bottom-up processing, cochlear implants, hearing impairment

## Abstract

The brain, using expectations, linguistic knowledge, and context, can perceptually restore inaudible portions of speech. Such top-down repair is thought to enhance speech intelligibility in noisy environments. Hearing-impaired listeners with cochlear implants commonly complain about not understanding speech in noise. We hypothesized that the degradations in the bottom-up speech signals due to the implant signal processing may have a negative effect on the top-down repair mechanisms, which could partially be responsible for this complaint. To test the hypothesis, phonemic restoration of interrupted sentences was measured with young normal-hearing listeners using a noise-band vocoder simulation of implant processing. Decreasing the spectral resolution (by reducing the number of vocoder processing channels from 32 to 4) systematically degraded the speech stimuli. Supporting the hypothesis, the size of the restoration benefit varied as a function of spectral resolution. A significant benefit was observed only at the highest spectral resolution of 32 channels. With eight channels, which resembles the resolution available to most implant users, there was no significant restoration effect. Combined electric–acoustic hearing has been previously shown to provide better intelligibility of speech in adverse listening environments. In a second configuration, combined electric–acoustic hearing was simulated by adding low-pass-filtered acoustic speech to the vocoder processing. There was a slight improvement in phonemic restoration compared to the first configuration; the restoration benefit was observed at spectral resolutions of both 16 and 32 channels. However, the restoration was not observed at lower spectral resolutions (four or eight channels). Overall, the findings imply that the degradations in the bottom-up signals alone (such as occurs in cochlear implants) may reduce the top-down restoration of speech.

## Introduction

Speech is commonly masked by background sounds in everyday listening environments. When portions of speech are masked by background sounds and thus not available to the auditory system, the brain may restore the missing segments using linguistic knowledge; syntactic, semantic, and lexical constraints; expectations; and context (Warren 1970; Warren and Sherman 1974; Bashford and Warren 1979; Verschuure and Brocaar 1983; Kashino 2006; Shahin et al. 2009), enhancing speech intelligibility (Warren 1983; Grossberg 1999; Sivonen et al. 2006).

One paradigm that is commonly used to quantify the effect of such top-down filling is phonemic restoration (PR), where better intelligibility of interrupted speech with periodic silent intervals is observed after these silent intervals are filled with loud noise bursts (Warren 1970; Warren and Obusek 1971; Warren and Sherman 1974; Bashford and Warren 1979; Verschuure and Brocaar 1983; Bregman 1990; Bashford et al. 1992; Kashino 2006). Huggins (1964) explained this finding from the point of bottom-up cues only; applying interruptions to speech introduces spurious cues, such as sudden offsets and onsets, which could potentially disrupt speech intelligibility. Adding a loud filler noise in the gaps makes these distortions inaudible. Also, Warren et al. (1994) suggested that the excitation caused from the noise could be reallocated to restore speech. Other studies demonstrated that listener expectation and linguistic and acoustic context can play important roles in PR (Warren and Sherman 1974; Warren 1983; Bashford et al. 1992; Sivonen et al. 2006; Grossberg and Kazerounian 2011). In short, PR seems to be a result of a complex interplay between bottom-up signal cues and top-down cognitive mechanisms.

Another observation that hints at the interactive nature of bottom-up cues and top-down mechanisms for PR is the connection between the continuity illusion and the PR benefit observed in increased intelligibility (Thurlow 1957; Repp 1992; Warren et al. 1994; Riecke et al. 2009, 2011). In a similar paradigm using simpler stimuli such as tones or noise, interrupted sounds combined with a filler noise can be illusorily perceived as continuous if there is no perceptual evidence against continuity. For example, noise bursts that have sufficient intensity and appropriate spectral content to potentially mask the sound segments induce a strong illusion (Warren and Obusek 1971; Bregman 1990; Kashino and Warren 1996). Additionally, it helps if there is no evidence of the interruptions when the interrupted speech is combined with the filler noise (Bregman 1990). When Bregman and Dannenbring (1977) introduced amplitude ramps on the tone signals at the signal–noise boundaries, the continuity illusion became weaker. Similar principles also apply to perceived continuity of speech (Powers and Wilcox 1977; Bashford and Warren 1979; Warren et al. 1994; Kashino 2006). Moreover, this illusory continuity percept of speech may help the cognitive auditory system to group the speech segments into an object, which in return may enhance speech intelligibility (Heinrich et al. 2008; Shahin et al. 2009). In fact, when amplitude ramps similar to those used by Bregman and Dannenbring (1977) were introduced at the speech–noise boundaries, Başkent et al. (2009) observed a reduction in perceived continuity of speech as well as a reduction in the PR benefit. These results suggest that when the bottom-up cues that are important for perceived continuity of speech are degraded, the PR benefit may be reduced.

The focus of the present study was on the degradation of bottom-up cues that may be caused by hearing impairment and/or front-end processing of hearing devices and how such degradation may affect phonemic restoration. These degradations would not be expected to affect perceived continuity of speech. We hypothesized that they may nevertheless reduce the PR benefit. In partial support of this idea, Başkent et al. (2010) have previously shown that elderly hearing-impaired listeners with moderate levels of sensorineural hearing loss may not benefit from PR. This population is also known to suffer disproportionately from background noise for understanding speech (Plomp and Mimpen 1979; Dubno et al. 1984; Schneider et al. 2005), and the reduced or non-existent benefit from PR could be a contributing factor. As PR strongly relies on top-down speech perception mechanisms, cognitive factors may play an important role in it. Therefore, it is logical to conclude that cognitive resources and skills changed by aging (van Rooij and Plomp 1990; Salthouse 1996; Wingfield 1996; Baltes and Lindenberger 1997; Gazzaley et al. 2005; Gordon-Salant 2005) might have caused the reduction of PR observed in this population. However, there is an alternative and more interesting hypothesis. The degradations in the signal quality due to peripheral hearing impairment might be the main reason for the reduced restoration benefit. This would be interesting because it would imply that the quality of the bottom-up speech signal, which may have nothing to do with the continuity illusion of interrupted speech, may still be important for the high-level speech perception mechanisms necessary for PR. The study by Başkent et al. was not designed to show these effects separately for age and bottom-up degradations due to hearing impairment, and additionally, it is difficult to find young listeners with typical forms of hearing impairment or elderly listeners with no hearing impairment (Echt et al. 2010; Hoffman et al. 2010). Therefore, their results only showed a reduction in PR with typical forms of hearing impairment of moderate to severe levels without exploring either hypothesis explicitly.

In the present study, we specifically aimed to test the alternative hypothesis mentioned above, namely, that the degradations in the bottom-up speech signal (in this study, due to simulated speech perception with cochlear implants (CIs)) may prevent the benefit from high-level top-down speech perception mechanisms (shown using PR). Two versions of noise-band vocoder processing were used to spectrally degrade speech: one that simulates speech perception with CIs and one that simulates speech perception with electric–acoustic stimulation (EAS), where acoustic low-frequency input from preserved residual hearing accompanies high-frequency electric input from the CI. By employing a young normal-hearing population, we have eliminated the potential effects of age and age-related cognitive changes. By using the vocoder processing, we have systematically varied the degree of degradations in speech stimuli in a manner that can occur in hearing-impaired listeners using CIs (Friesen et al. 2001). Moreover, because the vocoder processing makes the speech signals noisier, thereby making it easier for the listener to perceptually blend the speech segments with the noise bursts, we do not expect these degradations to counteract the continuity illusion of speech (Bhargava and Başkent 2011). Interrupted speech should theoretically be perceived as a continuous stream when combined with the filler noise under all degradation conditions, producing no direct bottom-up cue that would hint to interruptions in speech. The results, thus, will show only the effect of degradations in the bottom-up speech signal on the top-down repair mechanism of PR.

## Methods

### Participants

Sixteen young normal-hearing listeners (20 dB HL or better hearing thresholds at the audiometric frequencies of 250–8000 Hz), six men and ten women, with no history of hearing problems and between the ages of 17 and 33 years (average = 21.8 ± 5.5 years), participated in the study. All participants were native speakers of Dutch and students of the University of Groningen. Course credit was given for participation. All listeners were fully informed about the study procedure, and written informed consent was collected before participation.

### Stimuli

The PR benefit was quantified with a method that uses highly contextual sentences interrupted with periodic silent intervals (Powers and Wilcox 1977; Verschuure and Brocaar 1983; Başkent et al. 2010). In this method, speech recognition is measured once with the silent interruptions and once with these interruptions filled with loud noise bursts. The latter condition produces an illusory perceived continuity of interrupted speech, and additionally, an increase in intelligibility is observed, even though the noise bursts do not add speech information. The increase in intelligibility with the addition of noise is accepted as the objective measure for the PR benefit.

Speech stimuli of the present study were meaningful conversational Dutch sentences with high context (example: Buiten is het donker en koud [Outside is it dark and cold]), digitized at a 44,100-Hz sampling rate and recorded at the VU University Amsterdam (Versfeld et al. 2000). Versfeld et al. originally acquired the sentences in text from large databases, such as old Dutch newspapers. These were then pruned to only retain sentences with reasonable and comparable length, which represent daily-life conversational speech, are grammatically and syntactically correct, and semantically neutral. The resulting database has 78 balanced lists of 13 sentences, with 39 lists spoken by a male speaker and the other 39 by a female speaker. Each sentence is four to nine words long, containing words with up to three syllables.

All stimuli were processed digitally using Matlab. Two randomly selected lists, one spoken by the male talker and the other by the female talker, were used for each test condition. Sentences were first interrupted with silent intervals (with or without the filler noise). A speech-shaped steady noise, produced from the long-term speech spectrum for each speaker and provided with the recordings, was used as the filler. The interrupted sentences were then further manipulated using the noise-band vocoder processing (with reduced or full spectral resolution).

Interruptions were applied with a method similar to that of Başkent et al. (2010). The sentences and the filler noise were amplitude-modulated with a periodic square wave (interruption rate = 1.5 Hz, duty cycle = 50 %, 5-ms raised cosine ramp applied at onsets and offsets). A slow interruption rate was purposefully selected to induce the best PR effect based on previous studies (Powers and Wilcox 1977; Başkent et al. 2009, 2010). The modulating square wave started with the ON phase (amplitude = 1) for sentences and the OFF phase (amplitude = 0) for noise. The last noise burst was always played for the full duration, instead of adjusting to the sentence duration, to prevent the listeners from using potential timing cues that shorter duration noise bursts could provide. The RMS presentation levels were 60 and 70 dB SPL for sentences and noise, respectively, again selected based on previous studies (Powers and Wilcox 1977; Başkent et al. 2009, 2010).

Spectral degradations were applied to interrupted sentences (with or without the filler noise) with noise-band vocoder processing, a technique based on CI signal processing and commonly used to simulate speech perception with a CI (Dudley 1939; Shannon et al. 1995; Friesen et al. 2001; Başkent and Chatterjee 2010). First, the entire bandwidth of the processing was limited to 150–7,000 Hz. Then, spectral degradation conditions of 4, 8, 16, and 32 channels were implemented using a bank of bandpass filters (Butterworth filter, filter order 3). The cutoff frequencies of the filters were determined based on Greenwood’s mapping function (Greenwood 1990) by using equal cochlear distance, and the same cutoff frequencies were applied to both analysis and synthesis filters. Using half-wave rectification and low-pass filtering (Butterworth filter, filter order 3; cutoff frequency, 160 Hz), the envelopes were extracted from the analysis filters. Filtering white noise with the synthesis filters produced the carrier noise bands. The noise carriers in each channel were modulated with the corresponding extracted envelope. Adding the modulated noise bands from all vocoder channels produced the final speech stimuli. In a slightly different configuration, EAS was simulated by replacing the low-frequency channels of the vocoder with speech low-pass-filtered (LPF) at 500 Hz (Butterworth filter, filter order 3) in a manner similar to previous studies (Qin and Oxenham 2006; Başkent and Chatterjee 2010). For the reduced spectral resolution conditions of 4, 8, 16, and 32 channels, hence, the lowest one, two, four, and eight channels, respectively, were replaced with the unprocessed LPF acoustic speech.

### Procedure

The interrupted and spectrally degraded sentences were diotically presented using a Matlab Graphical User Interface through the S/PDIF output of an external Echo AudioFire 4 soundcard, a Lavry DA10 D/A converter, and Sennheiser HD 600 headphones. The listeners were seated in an anechoic chamber facing a touch screen monitor. A warning tone was played before each stimulus. The listeners were instructed to verbally repeat what they heard after each sentence was presented. Moreover, they were also told that they would hear only meaningful words in contextual and conversational sentences. Guessing and repeating only parts of the sentences were encouraged. With these instructions, even if some words were perceived ambiguously, the listeners had the chance to report what they thought they heard. These verbal responses were recorded with a digital sound recorder. When listeners were ready for the next presentation, they pressed the “Next” button on the screen. A student assistant who did not know the experimental conditions scored the recorded responses off-line. All words of the sentences were included in the scoring and no points were taken for wrong guesses. The percent correct scores were calculated by counting the number of the correctly identified words and taking its ratio to the total number of the words presented. These rules were followed in deciding correct identification: The propositions (de,het [the]) were only counted in scoring when the accompanied noun was also correctly reported. Confusions in personal pronouns (hij/zij [he/she]), demonstrative determiners (dit/dat [this/that]), past or present tenses of verbs (heeft/had [has/had]), singular or plural forms of verbs (heeft/hebben [has/have]), and diminutives (hond/hondje [dog/doggy]) were ignored, and these were counted as correct when reported in either form. If personal pronouns and demonstrative determiners were the only words reported from a sentence and if they were not entirely correct, then they were not accepted as correct. For compound words (vensterbank [windowsill]), reporting the partial words (venster [window] and bank [sill]) was not counted as correct. The same rule was applied to the verbs that are formed by adding prefixes to other verbs (vertellen [to tell] and tellen [to count]) as the meaning usually differed between such verbs.

The listeners of the present study were naive about the purpose of the experiment, and they had no prior experience with the sentence materials or the manipulations used in the experiment. A short training session was provided before actual data collection began using one list of uninterrupted sentences that were processed with a four-channel vocoder. The practice run was repeated with each listener until an intelligibility threshold of 30 % was reached (selected based on previous studies with similar materials and processing). There were four blocks of data collection (Table 1). The order of the blocks was counterbalanced using a Latin square design, and the order of the trials within each block was randomized. For each trial, one list of 13 sentences was used, and no sentence was played more than once to the same listener. No feedback was provided during training or data collection. The listeners were able to finish each block in 20–25 min and all blocks in one session. Including the explanation of the study and instructions, filling of the written informed consent, the audiometric test, and occasional breaks, one session lasted 3 h or less.

**TABLE 1:** Summary of the data collection blocks

Data collection block	Spectral resolution (no. of vocoder channels)	Speaker	Interruption	Vocoder simulation method	No. of trials	No. of sentences
1	4, 8, 16, 32, Full	Male	Silent or with filler noise	CI	5 × 2	130
2	4, 8, 16, 32	Male	Silent or with filler noise	EAS	4 × 2	104
3	4, 8, 16, 32, Full	Female	Silent or with filler noise	CI	5 × 2	130
4	4, 8, 16, 32	Female	Silent or with filler noise	EAS	4 × 2	104

CI refers to the vocoder simulation of cochlear implant processing; EAS refers to the second version of the vocoder simulation that was combined with unprocessed acoustic LPF speech, simulating combined electric–acoustic stimulation

## Results

**FIG. 1:**
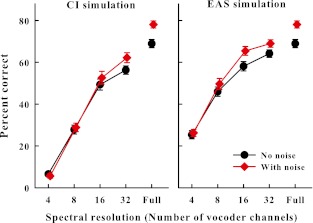
Average percent correct scores shown as a function of spectral resolution. The *error bars* denote 1 standard error. *Left panel* Results with vocoder processing (CI simulation). *Right panel* Results with vocoder processing combined with LPF acoustic speech (EAS simulation).

**FIG. 2.**
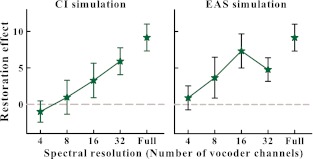
Improvement in speech performance with the addition of noise bursts, calculated from the difference in scores in Figure 1 and shown as a function of spectral resolution.

Figure 1 shows the percent correct scores, averaged across all participants and combined for stimuli spoken by the male and female speakers, as a function of spectral resolution. The left and right panels show the results with the two vocoder configurations, namely, CI and EAS simulations, respectively. The black circles and the red diamonds in each panel show the scores for stimuli with silent intervals or when the gaps were filled with the noise, respectively. The difference between the two scores for each spectral resolution condition shows the PR benefit, i.e., the improvement in intelligibility of interrupted speech after the noise bursts were added into the silent intervals. Figure 2 shows this PR benefit directly.

Figure 1 shows that reducing spectral resolution reduced speech intelligibility as all scores decrease with decreasing numbers of vocoder channels, for both configurations. Adding LPF speech to the vocoder (simulating an additional hearing aid) improved intelligibility as the scores in the right panel are better in general than those in the left panel. Both of these observations have been previously reported, but only with intelligibility of speech interrupted with silent intervals (Başkent and Chatterjee 2010). The main interest of the present paper was the effect of spectral degradation on the high-level perceptual mechanism of PR; therefore, we will focus our analysis on the restoration benefit, i.e., the improvement in scores after noise was added (the difference between the scores shown with diamonds and circles in Fig. 1 for each spectral resolution condition and the scores of Fig. 2). The rightmost data points in each panel in the figures show the baseline scores with interrupted speech before the vocoder processing was applied (full spectrum resolution). Hence, there was a baseline PR effect of 9 % before the interrupted sentences were spectrally degraded. Data show that the greater the spectral degradation was, the smaller was the PR benefit (Fig. 2). To analyze the significance of these effects, a two-factor repeated-measures ANOVA was applied with the within-subjects factors of number of vocoder channels and addition of noise for each vocoder configuration. The ANOVAs showed that there was a significant main effect of number of vocoder channels (*F*(4,60) = 533.96, *p* < 0.001, power = 1.00; *F*(4,60) = 370.40, *p* < 0.001, power = 1.00, for CI and EAS simulations, respectively), confirming that the performance decreased significantly as the spectral resolution decreased. There also was a significant main effect of adding the noise (*F*(1,15) = 8.10, *p* < 0.05, power = 0.71; *F*(1,15) = 28.20, *p* < 0.001, power = 1.00, for CI and EAS, respectively), confirming the PR benefit. The interaction was significant for the vocoder data shown in the left panel (*F*(4,159) = 5.52, *p* < 0.001, power = 0.93) and almost significant for the right panel (*F*(4,159) = 2.39, *p* = 0.061, power = 0.40), indicating that the PR benefit varied for different spectral resolution conditions. To further identify at what spectral resolution conditions a PR benefit was observed, a post hoc pairwise multiple comparison Tukey’s test was applied. This test showed that the baseline PR effect with full spectrum resolution (no vocoder processing) was significant (*p* < 0.001). When the spectral resolution was reduced the PR effect was significant only at the highest spectral resolution condition of 32 channels (*p* < 0.01) with the CI simulation, and at the spectral resolution conditions of 16 and 32 channels (*p* < 0.05 and *p* < 0.001, respectively) with the EAS simulation. At lower spectral resolutions, there was no significant benefit from restoration.

## Discussion

The main focus of the present study was on the bottom-up cues that could be degraded due to hearing impairment and/or front-end processing of hearing devices, and the effects of these degradations on the top-down speech repair mechanisms, quantified using the PR paradigm. The classical PR studies showed negative effects of bottom-up cue degradations on restoration when these degradations worked against perceived illusory continuity (e.g., Başkent et al. 2009). This is not entirely unexpected as a continuity percept is presumably related to the grouping of speech segments and the eventual object formation of interrupted speech, and such formation may contribute to the increased intelligibility (Heinrich et al. 2008; Shahin et al. 2009). Different from these studies, we hypothesized that the hearing impairment and/or hearing device-related degradations in bottom-up cues could also reduce the restoration benefit, even though they were not expected to work against perceived continuity (Bhargava and Başkent 2011). The PR benefit observed in the present study was thus assumed to be primarily a measure of top-down repair (e.g., Sivonen et al. 2006). As a result, the study emphasizes that the top-down repair may not function adequately when substantial degradations are present in the bottom-up speech signals, and this may additionally contribute to speech perception problems that the hearing-impaired listeners have in challenging real-life listening situations.

In line with the present study’s hypothesis, Başkent et al. (2010) had previously shown that the benefit from PR was reduced or absent in moderate levels of sensorineural hearing loss. However, in this study, the degree of hearing loss and the age of the participants co-varied; the listeners with more hearing loss were also older in general. As cognitive resources may decline with aging (e.g., Salthouse 1996; Wingfield 1996; Gordon-Salant 2005), and due to the importance of cognitive mechanisms for PR, age could potentially have contributed to the reduced PR benefit observed in the study by Başkent et al. In the present study, the effect of bottom-up signal degradations on PR benefit was studied for another form of degradation, reduced spectral resolution, which could occur with hearing-impaired people, specifically with users of CIs. By simulating implant processing and testing young normal-hearing listeners, the potential effects from age and age-related changes in cognitive resources were entirely eliminated. Because noise-band vocoder processing produces stimuli that are more noise-like in quality, we assumed that vocoded speech segments would be easier to be perceptually bound with filler noise bursts, strengthening the perceived continuity. Hence, this degradation was not expected to work against object formation per se. Additionally, the spectral degradation could be applied systematically by varying the number of the vocoder processing channels. This systematic approach was expected to produce a gradual reduction in the restoration benefit so that the limit of the top-down repair could be explored. In line with our expectations, the results with the vocoder simulation of CIs showed that there was a benefit from PR only in the highest spectral resolution condition of 32 channels. All lower spectral resolution conditions, 16 channels or less, prevented the PR benefit.

A second simulation was also implemented in the present study to simulate speech perception with EAS. Adding acoustic low frequencies to the electric perception through the CI has been shown to help speech intelligibility in adverse listening conditions (Kong and Carlyon 2007; Büchner et al. 2009; Başkent and Chatterjee 2010). One of the advantages that EAS provides is the addition of strong voicing information (Brown and Bacon 2009; Cullington and Zeng 2010; Zhang et al. 2010), a cue important for speech perception, especially in complex auditory scene analysis with interfering background sounds (Bregman 1990). However, voicing pitch is not fully delivered with CIs (Gfeller et al. 2002; Qin and Oxenham 2006). This cue is also considered to be important for binding sound segments that are audible through distortions, such as fluctuating noise or temporal interruptions (Grossberg 1999; Plack and White 2000; Başkent and Chatterjee 2010; Chatterjee et al. 2010). Because a continuity percept seems to be important for the restoration of interrupted speech (Powers and Wilcox 1977; Bashford and Warren 1979; Heinrich et al. 2008; Başkent et al. 2009; Shahin et al. 2009), a strong voicing cue could conceivably provide better benefits from PR as well. The results of the present study indeed showed that there was a slight improvement in restoration benefit in the EAS simulation compared to the CI-only simulation. While with the CI simulation, a spectral resolution of 32 channels was required for PR to be observed; once the unprocessed LPF speech was added, a spectral resolution of 16 channels was also sufficient. However, at lower spectral resolutions, even though the overall scores were higher with the EAS (as shown by the comparison of the scores in the right panel to the scores in the left panel of Fig. 1), restoration benefit was still not observed.

While the present study indicates a deficit in PR as a result of spectral degradations, it does not specify what specific factors have contributed to this finding. Inspecting Figure 1, one could conclude that a certain amount of speech intelligibility is required (above 50 % in this case) to be able to restore as the PR effect was only observed for these levels of intelligibility. However, earlier data by Verschuure and Brocaar (1983) showed that, with normal-hearing listeners and using interrupted speech that was not degraded otherwise, PR benefit was actually larger when speech intelligibility was lower. Reanalyzing data from an earlier study (Başkent et al. 2010), Başkent (2010) later showed that even at a high baseline speech intelligibility, no restoration was observed with moderately hearing-impaired listeners, ruling out this potential explanation. Hence, a low-level intelligibility of interrupted speech alone does not seem to be the cause of reduced PR benefit.

However, the factors that cause low intelligibility could affect the PR differently for degraded and non-degraded speech (or between hearing impairment and normal hearing). Some such factors are relevant to speech intelligibility in general while also relevant to restoration. For example, glimpsing of speech through noise segments (Barker and Cooke 2007) and integration of these speech segments into an auditory object (Shinn-Cunningham and Wang 2008) can be affected by peripheral degradations. Integration imposes an interesting problem in the context of vocoder simulations and CIs. The vocoded speech segments are noisy in nature and therefore should be easier to integrate with the noise segments. Assuming an overlap between perceived continuity and the top-down repair of degraded speech (Heinrich et al. 2008; Başkent et al. 2009; Shahin et al. 2009), theoretically, a better PR benefit could be observed. However, this was not the case in the present study. Hence, further research is needed to identify what specific factors play a role in the restoration of interrupted and spectrally degraded speech.

The results of the present study fit well within the general research area of how degradations in bottom-up speech signals may have effects on a variety of top-down processes. For example, Sarampalis et al. (2009) showed that speech presented in background noise increased listening effort, indicated by longer reaction times in a dual-task paradigm, even when there was no change in intelligibility. Other studies focused on signal degradations due to hearing impairment. Rabbitt (1991) observed that elderly hearing-impaired listeners had problems remembering words presented to them auditorily, even though they could understand and repeat back these words. More recently, Aydelott and Bates (2004), Aydelott et al. (2006), and Janse and Ernestues (2011) showed that the semantic facilitation from context was reduced with both elderly hearing-impaired listeners and young listeners who were tested with simulations of hearing impairment. Shinn-Cunningham and Best (2008) argued that peripheral degradations caused by hearing impairment could negatively affect selective attention and object formation, and Hafter (2010) similarly argued for potential negative effects of hearing device processing on cognitive processes. These studies, combined, point to the strong connection between top-down processes and the effects of bottom-up degradations in speech signals caused by hearing impairment, aging, or other distortions on these processes. Furthermore, one can conceive that other factors, such as increased listening effort, reduced memory, semantic facilitation from context, or selective attention, may negatively affect the cognitive resources needed for top-down repair.

Overall, from a scientific point of view, the findings of the present study further emphasize the crucial interaction between the bottom-up speech signals and their top-down interpretation and enhancement for robust speech perception, especially for speech distorted due to noise or hearing impairment (Grossberg 1999; Alain et al. 2001; Grimault and Gaudrain 2006; Pichora-Fuller and Singh 2006; Zekveld et al. 2006; Davis and Johnsrude 2007). From a practical point of view, the results are potentially important for hearing-impaired listeners with CIs. For speech intelligibility, the vocoder simulation with eight spectral channels has been shown to be functionally closest to a typical CI user (Friesen et al. 2001). With eight vocoder channels, in the present study, there was no PR benefit with the CI simulation. More surprisingly, however, with eight channels, there was also no PR benefit with the EAS simulation. This is partially in contrast with the previous studies that showed an advantage from the added unprocessed low-frequency acoustic speech. For example, Qin and Oxenham (2006) and Kong and Carlyon (2007) showed an improvement in intelligibility of (uninterrupted) speech in noise with similar simulations after unprocessed low-frequency speech was added to the vocoder. With a similar manipulation, Başkent and Chatterjee (2010) recently also showed better perception of interrupted speech (with periodic silent intervals) with EAS over CI simulation. This improvement was largest for the lowest spectral resolution conditions of four and eight channels. The perception of speech interrupted with silence intervals is one of the conditions of the present study as well. The comparison between the filled circles in the left and right panels of Figure 1 shows an improvement due to EAS, similar to that reported by Başkent and Chatterjee (2010). However, this improvement in baseline scores with interrupted speech was not reflected in an improvement of the PR benefit (Fig. 2). Hence, while the additional cues provided by the unprocessed LPF speech were useful to enhance one form of restoration, i.e., the perceptual restoration of speech interrupted with silence intervals, they were not sufficient for the other form of restoration, i.e., the top-down filling when the loud noise bursts were added. A recent study by Benard and Başkent (under review) explored perceptual learning with the two forms of interruptions: one with silence and the other with silent intervals filled with noise. As they observed similar learning trends with the two forms, they concluded that similar cognitive mechanisms should play a role in the restoration of speech with silent intervals and speech with noise-filled intervals. However, the present study implies that the restoration of the latter may be more sensitive to the bottom-up signal degradations.

All results combined, thus, show that the top-down repair mechanisms have limitations, and as the degradations in bottom-up speech signals increase, these mechanisms may fail to be helpful. Thus, the speech intelligibility difficulties that CI users encounter in interfering background noise could partially be caused by the top-down repair mechanisms that do not function properly as a result of the degraded input from the periphery. The current speech intelligibility tests used in the clinical diagnostic and rehabilitation procedures are not yet designed to show the effects from high-level speech perception mechanisms (or their failure); therefore, such deficiencies may be difficult to identify in the clinical settings. For future research, our results imply that increasing the fidelity of the speech signals processed and sent to the electrodes may also increase the functional use of top-down processing for better speech perception in CI users.
